# Patient, surgeon, and healthcare purchaser views on the use of decision and communication aids in orthopaedic surgery: a mixed methods study

**DOI:** 10.1186/1472-6963-14-366

**Published:** 2014-08-31

**Authors:** Kevin J Bozic, Kate Eresian Chenok, Jennifer Schindel, Vanessa Chan, James I Huddleston, Clarence Braddock, Jeffrey Belkora

**Affiliations:** Department of Orthopaedic Surgery, University of California, 500 Parnassus, MU 320W, San Francisco, CA 94143-0728 USA; Philip R. Lee Institute for Health Policy Studies, University of California, 3333 California Street, Suite 265, Box 0936, San Francisco, CA 94118 USA; Pacific Business Group on Health, 221 Main Street, Suite 1500, San Francisco, CA 94104 USA; Healthcare Research, Strategy and Design, 2423 Byron Street, Berkeley, CA 94702 USA; Department of Orthopaedic Surgery, Stanford University Medical Center, 450 Broadway Street, MC 6110, Redwood City, CA 94063 USA; David Geffen School of Medicine, University of California, 10833 Le Conte Avenue, 12–138 CHS, Box 951722, Los Angeles, CA 90095-1722 USA

## Abstract

**Background:**

Despite evidence that decision and communication aids are effective for enhancing the quality of preference-sensitive decisions, their adoption in the field of orthopaedic surgery has been limited. The purpose of this mixed-methods study was to evaluate the perceived value of decision and communication aids among different healthcare stakeholders.

**Methods:**

Patients with hip or knee arthritis, orthopaedic surgeons who perform hip and knee replacement procedures, and a group of large, self-insured employers (healthcare purchasers) were surveyed regarding their views on the value of decision and communication aids in orthopaedics. Patients with hip or knee arthritis who participated in a randomized controlled trial involving decision and communication aids were asked to complete an online survey about what was most and least beneficial about each of the tools they used, the ideal mode of administration of these tools and services, and their interest in receiving comparable materials and services in the future. A subset of these patients were invited to participate in a telephone interview, where there were asked to rank and attribute a monetary value to the interventions. These interviews were analyzed using a qualitative and mixed methods analysis software. Members of the American Hip and Knee Surgeons (AAHKS) were surveyed on their perceptions and usage of decision and communication aids in orthopaedic practice. Healthcare purchasers were interviewed about their perspectives on patient-oriented decision support.

**Results:**

All stakeholders saw value in decision and communication aids, with the major barrier to implementation being cost. Both patients and surgeons would be willing to bear at least part of the cost of implementing these tools, while employers felt health plans should be responsible for shouldering the costs.

**Conclusions:**

Decision and communication aids can be effective tools for incorporating patients preferences and values into preference-sensitive decisions in orthopaedics. Future efforts should be aimed at assessing strategies for efficient implementation of these tools into widespread orthopaedic practice.

**Electronic supplementary material:**

The online version of this article (doi:10.1186/1472-6963-14-366) contains supplementary material, which is available to authorized users.

## Background

Recent policy work on informed patient choice proposes that patients should achieve a state of adequate knowledge about their medical condition, ask questions and involve themselves in treatment decisions, and act on well-considered preferences [1]. Decision and communication aids are decision support interventions aimed at achieving these goals. Decision aids consist of print or audio-visual materials that inform patients about the risks and benefits of a specific crossroads, such as the decision about whether or not to have surgery [2]. Unlike traditional patient education materials, decision aids always present the risks and benefits of simply foregoing further medical intervention as a baseline for comparison. Decision aids therefore address patient needs for orienting information. Communication aids include question lists [3], audio-recordings [4, 5], and after-visit summaries [6], which can be packaged into an integrated intervention delivered by a health coach [7], along with decision aids [8, 9]. Communication aids effectively address patient needs to rehearse their questions and concerns and review the content of discussion with the care team [10, 11].

Despite evidence that these tools are effective for enhancing decision quality for preference sensitive conditions and are associated with appropriate, patient-centered care [3, 5, 12, 13], their adoption in the field of orthopaedic surgery has been limited [1, 14, 15], with a few notable exceptions. A group of investigators from the Group Health Cooperative reported high satisfaction among all stakeholders and reduced utilization of surgery and overall costs after implementation of decision aids for patients with hip and knee arthritis [16, 17]. Many healthcare stakeholders, including payors and policymakers, have promoted reduction in utilization of elective surgical procedures as one of the primary benefits of exposing patients to decision aids and related decision support interventions. This message could lead to conflict among healthcare stakeholders as reduced surgery rates are attractive to payers but could be barriers to adoption by surgeons or patients if they see the interventions as attempting to persuade them to avoid surgery, or to ration care or minimize costs. However, the Group Health study was a non-randomized study, and the subset of patients who actually *received* decision aids elected to have surgery *more* frequently than those who did not. Moreover, a separate randomized study incorporating decision aids and coaching along with other care management strategies showed overall reductions in utilization and cost [18], but out of 6 preference sensitive conditions (including hip and knee osteoarthritis) only one (heart disease) showed statistically significant reductions in rates of surgery; and the overall cost savings could have been attributable to that one condition, or even to better management of patient comorbidities such as diabetes or other chronic conditions [19].

In order to address these and other controversies and gaps in the literature, we conducted a three-year, multi-phase investigation of the use of decision and communication aids in patients with advanced osteoarthritis of the hip or knee. Our goals were to assess the impact of these interventions on the efficiency and effectiveness of decision-making; and to assess patient, surgeon and healthcare purchaser perceptions of these interventions. Regarding efficiency and effectiveness, we previously completed a randomized controlled trial (RCT) to assess the effectiveness of decision and communication aids and reported that patients with hip and knee OA who were randomized to receive decision and communication aids arrived at an informed decision during the first visit more often than patients who did not receive them [20], with no difference in utilization of surgery. We interpreted this as evidence that decision and communication aids can promote effective and efficient patient choice in orthopaedic practice.

The purpose of this mixed-methods study was to evaluate the perceived value of implementing decision and communication aids in orthopedic practice. Specifically, we assessed experience with and level of interest in decision and communication aids and willingness-to-pay for decision and communication aids among patients, surgeons, and healthcare purchasers.

## Methods

### Patients

To assess patient perceptions, we surveyed a subset of patients who received decision and communication aids in our aforementioned RCT (n = 26) [20]. These patients were randomly chosen from the patients who completed our online follow up survey, patients were sorted by last name, and a random number generator and the first 15 people from each study site, 30 people total were selected to be invited to participate in a telephone interview, approximately 12 months after their initial clinic visit. Of the 30 people invited, 26 patients completed the follow up survey. We invited these patients to participate in an online survey about what was most and least beneficial to them about each of the interventions they received: a decision aid, a question-listing session, a recording of the consultation, and a copy of the physician’s dictated note. We also asked about the ideal mode of administration of these tools and services, and their interest in receiving comparable materials and services in the future.

Of the 26 patients who received decision and communication aids in the RCT who completed this survey, 13 patients (6 from one center and 7 from the other center) agreed to further participate in a telephone interview in which they were asked to 1) provide a baseline ranking of importance (scale of 1–5) for each of the decision and communication aids (DVD/booklet, question-listing and post-visit recording/notes) and 2) attribute a monetary value to varied groupings of these elements reflecting their hypothetical willingness-to-pay for these tools along with their rationale. Interviews were audio-recorded and written transcripts, notes and data from patient responses were thematically coded and interview transcripts and field notes were analyzed using Dedoose qualitative and mixed-methods analyses software [21] (Dedoose Version 4.5, web application for managing, analyzing, and presenting qualitative and mixed method research data (2013). Los Angeles, CA: SocioCultural Research Consultants, LLC (http://www.dedoose.com)). Written informed consent was obtained for all participants who participated in a phone interview.

### Physicians

Our RCT found that surgeons rated their interactions with intervention patients as more effective and efficient than those with control patients [20]. In order to assess perceptions of decision and communication aids in the broader orthopedic surgeon community, we developed and fielded an online and written survey distributed to 1,290 members of the American Academy of Hip and Knee Surgeons (AAHKS) (Additional file 1), the largest professional association of hip and knee surgeons in the U.S*.* We received 518 responses, a response rate of 40%.

### Healthcare purchasers

In order to assess healthcare purchaser interest and involvement in promoting use of decision and communication aids, we reviewed relevant literature, including the most recent Cochrane systematic reviews on the impact of such tools [10, 11, 22], interviewed employer members of the Pacific Business Group on Health (PBGH), and interviewed individuals who are knowledgeable about purchaser perspectives on patient-oriented decision support. We used themes from our research and interviews to develop a written survey that we distributed to 12 employer members of PBGH in December 2012. Individuals representing six of the employers responded, a response rate of 50%. The industries represented among the companies included Energy, Government, Aerospace, Higher Education, Insurance, Retail, Information Technology, Utility, Technology and Automotive. The number of individuals employed by each company ranged from 3,000 – 1.3 million.

We received IRB approval from the University of California, San Francisco, Human Research Protection Program Committee on Human Research.

## Results

### Patients’ perceived value of decision and communication aids

Of the patients who received the intervention materials or services and completed the follow-up survey, the majority reported they would want to receive similar materials or services in the future. Patient satisfaction was consistent across all intervention elements: 77% (20/26) would want to receive a similar decision aid (DVD/booklet); 76% (19/25) would want to receive a question-listing service; 83% (20/24) would want to receive notes from their visit; and 80% (19/24) would want to receive a recording from their visit in the future.

Open-ended survey responses about what patients valued most about the specific interventions indicated that a) the decision aid provided helpful information and stimulated patient questions; b) the question-listing helped patients gain clarity on their own thoughts, questions and concerns and feel prepared for their visit with their doctor; and c) the dictated notes and CD recording of the visit were helpful for patients to review after their visit and to share with others (e.g., family members) [Table 1].

**Table 1:** **Patient-reported benefits of decision and communication aids**

DVD	Total responses = 23
Provided information – detailed, overview and/or easy to understand	7 (30%)
Helped patients prepare and generate questions	4 (17%)
Showed the MD was making an effort	1 (4%)
Helped show the patient point of view	1 (4%)
Could not recall	7 (30%)
N/A	3 (13%)
**Question-listing**	**Total responses = 21**
Helped patients gain clarity of own thoughts/questions/concerns	9 (43%)
Felt prepared	3 (14%)
Provided information	3 (14%)
Felt informed	1 (5%)
Health coach was ally/good listener	1 (5%)
Could not recall	2 (10%)
N/A	2 (10%)
**Written copy of dictated notes**	**Total responses = 15**
Can review/access information from surgeon visit	6 (40%)
Can share information with others	3 (20%)
Can verify information discussed	2 (13%)
N/A	4 (27%)
**CD recording of office visit discussion**	**Total responses = 12**
Can review/access information from surgeon visit	7 (58%)
Can share information with others	1 (8%)
Provided detailed information	1 (8%)
N/A	3 (25%)

Follow up telephone interviews provided further insights into patients’ hypothetical willingness-to-pay for these materials and services. While survey results discussed above indicated a high level of satisfaction and desire to have these materials in the future, in-depth interviews revealed mixed messages among patients for paying for decision and communication aids.

Among the 13 interview respondents, all of whom had received all the decision and communication aids as part of the previous randomized controlled trial, 10 (77%) were willing to pay something for access to one or more of the tools. The remainder (3) stated they would not be willing to pay anything for any of these tools. We asked about willingness to pay for graduated levels of decision and communication aids. The median willingness to pay started at $10 for decision aids only, rose to $25 for decision aids plus question listing, and rose again to $50 for decision aids plus question-listing plus notes and recordings. However, for each scenario, half the patients reported no willingness to pay [Table 2].

**Table 2:** **Patients willingness-to-pay for decision and communication aids (N = 13)**

Willingness to pay	DAs only	DAs and question-listing	DAs, question-listing, and recording/notes
Mean	$31	$65	$87
Median	$10	$25	$50
Mode	$0 (5/13)	$0 (6/12)	$0 (6/13)

The in-depth interviews revealed that patients who were more willing to pay some dollar amount for these materials tended to evaluate the broad impact of these tools when considering their value by focusing on the benefits of the materials as a collective whole, finding particular benefit in the continuity that the tools provided pre and post visit:

*“…the connection between let’s say my first visit…we reviewed or looked at some of the materials or some of my responses I think or questions or concerns. And that was good …making sure there’s a continuum between all that preceding questioning and the first visit.” (J7A29)*

Their responses indicted they were conceptually assessing value the tools in terms of their overall *value*, how the elements collectively impact their decision-making experience and serve as a kind of public good for patients:

*“It was extremely valuable because you gain the confidence to ask the right, hard questions where you may not have your thoughts gathered, you may be intimidated…that pre-planning session really helps you focus…it was crucial to my sense of confidence, satisfaction, comfort level with going in and having this done. The back side of it, having the transcript and the CD of all of this was also really very satisfying from a gut level in that obviously care and attention was taken… what was done there was extremely valuable, it’s something tangible that’s in your hand, you can share it with somebody.” (J7K24)*

Those patients who were more reluctant or adamant about not paying anything for these tools tended to default to a more narrow and individualized personal cost-benefit analyses in which they considered the fact that they may not “need” the materials enough to pay for them and could conduct their own internet research or just ask their doctor questions when they have them:

*“I wouldn’t pay anything for it, just because, again, I’m knowledgeable on the medical subject and if I have questions, I research it online. You know, through the medical journals or Medscape or something like that. I mean, out of those three materials, I did like the booklet, I thought it was really good, I didn’t like the section on the drug write-up. But, you know, I did like that, I thought that book was really good.” (D8M27)*

*“I mean, not that I can’t afford it or anything, I think I would just wait until I found a doctor and ask the questions there.” (A8F27)*

When prompted to consider their willingness to pay, some patients tended to devalue and distance themselves from the potential benefit of the tools if they did not end up having joint replacement surgery. They focused on the fact that the materials did not always speak directly to their personal clinical situation and retrospectively assessed the material with additional scrutiny in considering their own willingness to pay.

*“What this meant was, here we are with a hip which is already destroyed and now eventually after everything was healed, they have to get in there and fix the destroyed hip, it’s a two-step operation…nothing like that showed up in the DVD. It was very different than most of the happy people in the DVD and, you know, they didn’t have this situation.”* (C9V26)

*“So all my hesitation, in general, with this is… yeah it’d be worth 50 bucks, it’d be worth $100, the big caveat to me would be… I’d feel like you’re telling me about stuff that I’m gonna need in 20–30 years, you know, and it doesn’t apply to me now. As shocked as I was when watching the knee replacement, the important thing was that they had people in there who decided to do some strengthening and not get the knee replacement which is what we did, and how they lived their life from that point. That information was what I wanted for my situation…” (B9W12)*

A few patients explained that their lack of willingness-to-pay was based on the simple fact that this kind of offering should be a part of standard medical care:

*“So I guess, I’m thinking that anything to do with medical should be included with the Medicare.” (D9C16)*

*“No, and I could afford all of these numbers. I’m probably gonna’ say no, because I think that preparing the patient for the experience is part of the medical service. So, making it some “extra”, like, I go to the dentist and they clean my teeth and then they say “well do you want a tooth whitening kit?” That’s a different service. But, I think preparing the patient for the surgery is part of the medical service being offered. (N8M15)*

### Surgeons’ experience with decision and communication aids

The majority of surgeons surveyed do not routinely provide their patients with decision aids that explicitly cover surgical and non-surgical treatment options for OA of the hip and knee prior to or following their visit with their surgeon. Of the 518 surgeons surveyed, 31% do not provide educational materials on the risks and benefits of surgery, 14% do not provide educational materials on non-surgical options, 22% do not have patients develop questions prior to their visit with their surgeon and 27% do not provide patients with dictated notes or a CD recording summarizing their visit and treatment discussion [Table 3]. Surgeons do access informational content to distribute to patients, primarily through their medical group or hospital system (40%) and/or by purchasing materials directly through vendors (27%). A small percentage of surgeons reported they develop their own materials (16%) and/or direct patients to information via handouts or websites such as AAOS/Ortho Info and AAHKS.

**Table 3:** **Current use of educational materials among orthopaedic surgeons**

Do routinely provide educational materials on benefits/risks of surgery prior to office visit	31%
Do routinely provide educational materials on non-surgical options prior to office visit	14%
Do have patients develop questions prior to visit	22%
Do provide patients with notes or CD recordings summarizing visit and treatment discussion	27%

**Figure 1:**
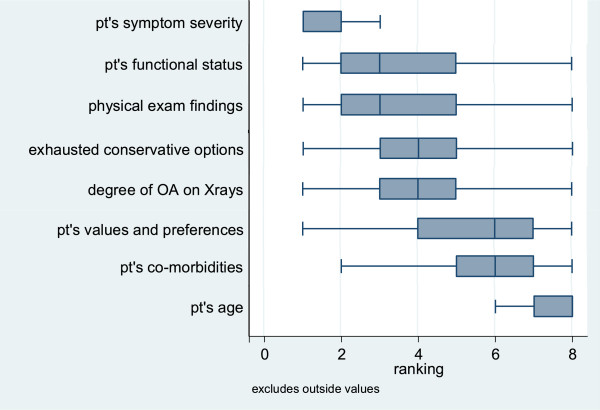
**Factors that affect recommendations regarding hip or knee replacement.** “1” factor that most strongly affects your recommendations. “8” factor that least affects your recommendations.

Although health services researchers consider osteoarthritis to be a preference-sensitive condition [23], meaning that patient preferences should weigh heavily in treatment decisions, surgeons ranked “patient values and preferences” among the least important factors in a list of eight factors that influence their recommendations regarding hip or knee replacement [Figure 1]. When asked *how* they assess patients’ preferences about surgical versus non-surgical treatment options, approximately half of surgeons’ responses (51%) included a general reference to asking, discussing or talking to patients, with 20% of responses including references to talking with patients during their assessment and discussion of various clinical factors such as x-rays, physical exam, patient history/treatment, response to prior treatment, symptom severity/quality of life, functional status. Only 14% of surgeons’ responses included a reference to directly soliciting and addressing patients’ preferences, expectations about treatment outcomes and/or knowledge of their condition and treatment options.

### Surgeons’ perceived value of decision and communication aids

While the majority of surgeons surveyed do not routinely use formal decision aids or communication aids, they do perceive value in these tools, particularly for patients. Results show that 78% believe decision aids would increase patients’ satisfaction and slightly less (68%) believe communication aids would increase patients’ satisfaction. In terms of how these tools impact surgeons more directly, 70% believe decision aids would increase the quality of their interactions with patients and 56% believe communication aids would do so as well. All surgeons believe the use of decision and communication aids would have a neutral effect or better effect on their professional satisfaction.

While surgeons believe decision and communication aids could have some positive impact, they do not readily perceive these tools to be something they would use to market the quality of their care more broadly. Surgeons were somewhat split in terms of whether they would market decision aids and communication aids as an indicator of quality of care to patients and payers with just under half (49%) saying they would use decision aids to market quality of care and 40% saying they would use communication aids to market quality of care.

In order to have surgeons assess a monetary value of these tools and estimate the cost impact on their practice, we presented a hypothetical package of decision and communication tools that were proven to lead to more productive visits and had no effect on visit length. When asked what they would be willing to pay for these tools in their practice, surgeons indicated they would be willing to pay on average $7.50 per patient for the use of these tools. Surgeons expected to cover these costs primarily through increased productivity (50%) and/or increased volume of patients (42%). Some surgeons indicated they expected to pay for these tools through a loss (14%), which they described in terms of absorbed costs, increased overhead, loss in revenue, and/or out of pocket/decreased income.

### Surgeons’ perceived impact of decision and communication aids on surgery rates

The majority of surgeons surveyed do not expect decision aids and communication aids would have an impact on surgery rates. Most think decision aids and communication aids would have a neutral effect on patients choosing surgery (81% and 82% respectively), while the remaining small percent of surgeons think it would increase the frequency of patients choosing surgery. Almost no surgeons think the use of these tools would decrease surgery rates.

### Purchasers’ views of decision and communication aids

**Figure 2:**
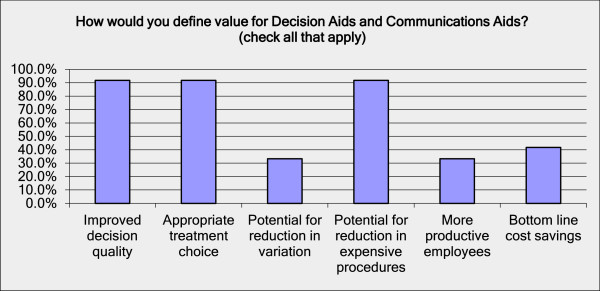
**Purchaser’s definition of value for decision and communication aids.** Note: multiple responses allowed. N=12.

The environmental scan and survey of large employers confirmed that purchasers have a wide range of patient-oriented programs that are similar to or complementary to decision and communication aids. These programs include health education materials, nurse advice lines and second opinion services. Purchasers offer these programs because they believe that they improve decision-quality and treatment choice and also because they think they have the potential to reduce utilization of expensive, unnecessary procedures. Fewer define bottom line cost savings, potential reduction in variation and more productive employees as the primary value of these tools [Figure 2]. Purchasers surveyed offer a number of tools and programs to help with medical decision-making, but few offer their employees an integrated program that includes decision and communication aids. Most purchasers provide health and disease-specific education materials, cost calculators, and other decision support tools. Most frequently, these tools are provided via health plan portals. Many purchasers also provide access to health coaching, most often provided via their health plans. These patient engagement programs provide the opportunity to reach patients at many points in the referral and decision process, rather than only at the point of surgical intervention. For patients who are already at a point of surgical decision, some purchasers also offer second opinion services provided by a third party vendor.

PBGH members surveyed who do offer integrated decision support tools either purchase them directly from a vendor, such as Health Dialog, Healthwise, or Emmi Solutions, or have these programs available to them through their health plans. Purchasers surveyed believe decision support is part of the provision of appropriate care, and that it should be included in standard workflows.

## Discussion

### Analysis and interpretation of findings and connections to the literature

**Figure 3:**
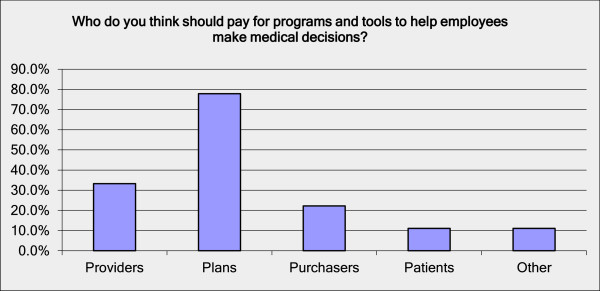
**Purchaser’s opinions on who should pay for programs and tools to help employees make more informed medical decisions.** Note: multiple responses allowed. N=12.

Our findings suggest that patients, surgeons, and healthcare purchasers all recognize the potential value of decision and communication aids in orthopaedics. Their only major concern was who should pay for them. Patients surveyed in this study found decision and communication aids to be helpful, and did not report the barriers reported by other investigators [24], but were split in their willingness to pay. The median willingness to pay of $50 for the full package of decision and communication aids was lower than the $150 willingness to pay for question-listing alone found in a study among patients in a rural setting [25]. Similarly, surgeons perceive value in using decision and communication aids, but expressed concern about bearing the costs associated with their implementation. This is similar to findings in surveys and other studies of health professionals, as summarized in a systematic review, except that our study did not find concerns about lack of applicability of these tools for patients [26]. A survey similar to ours conducted in Hawaii found that physicians also reported low use of decision aids and expressed reservations about lack of resources for implementation [27]. Similar to a recent implementation study that included orthopedics, we found other concerns about the time and effort required to adopt and implement such tools [17], but generally decision and communication aids were not seen as threats to surgery rates, professional livelihood, professional autonomy, patient anxiety, or other such potentially challenging issuesPurchasers recognized that decision and communication aids could help activate patients to be more involved in their own care, but most felt that health plans should be responsible for absorbing the costs associated with implementing them as part of the cost of delivering care [Figure 3].

Decision and communication aids have been promoted by policymakers as a means of engaging patients and their caregivers more directly in their medical decision making, with the goal of more closely matching treatment decisions with patients preferences and values and reducing inappropriate utilization of invasive surgical procedures. The field of orthopaedic surgery, which has been characterized as ‘preference sensitive care’, would seem ripe for such interventions. However, despite the widely reported benefits in terms of improving patient knowledge and decision quality [28–32], decision and communication aids are rarely used in routine orthopaedic practice.

Going forward, in order to facilitate widespread adoption of decision and communication aids into orthopaedic practice, each of the stakeholder groups interviewed must be willing to make changes to the way they deliver, receive, and pay for care. Patients must be willing to spend time educating themselves on their disease process and treatment options in advance of the visit, as well as explicitly share their preferences and values with their healthcare team. Surgeons must be willing to actively consider their patients’ preferences and values when formulating a treatment plan. One of the most interesting findings in our study was the fact that surgeons ranked “patient preferences and values” among the least important factors they consider when deciding whether or not joint replacement surgery is appropriate for a patient with advanced arthritis of the hip or knee. Given that joint replacement surgery is by definition a “preference sensitive” procedure, it would stand to reason that understanding patient preferences and values would be important when devising a treatment plan. Our finding may be an indication that surgeons lack a formal mechanism for eliciting patient preferences and values; as shown in our RCT, soliciting a question list from an informed patient could be an efficient way for surgeons to gather this information.

Finally, health plans and purchasers would need to help facilitate adoption of decision and communication aids by offering novel benefit designs and other incentives to patients and providers who take the time to participate use these tools. Of note, PBGH members surveyed in our study and purchasers interviewed in other studies see benefit designs as an important lever in encouraging patients to become more informed and involved and incenting providers to offer decision and communication aids [Table 4]. For example, a purchaser could offer plans with differential cost-sharing where a patient could access a lower deductible, lower copayment, or lower coinsurance value if they offer proof of using decision and communication aids. Other, simpler financial incentives could be rewards, such as gift cards, or monetary contributions to health savings accounts for patients who use decision and communication aids.

**Table 4:** **Informed choice tools that purchasers are willing to pay for**

Tools	Total responses = 10
Benefit design to incent use of cost/quality	10 (100%)
Price comparisons and calculator tools	10 (100%)
Third party vendors for second opinions	8 (80%)
Narrow networks to drive equality	8 (80%)
Educational materials	7 (70%)
Tools to help patients develop questions for their physicians	6 (60%)
Formal SDM programs	5 (50%)

Purchasers did not rate “bottom line cost savings” among the primary benefits of decision and communication aids. Nor did surgeons express concerns about loss of revenue due to reduced utilization. These are encouraging findings that suggest there is an opportunity to find common ground among stakeholders with regards to the role and benefits of decision and communication aids in orthopedics.

Both state and federal health care reforms have accelerated a move to value-based payments. In these programs, providers may accept a fixed fee for an entire episode or care or for the health of specific individuals. These programs, which hold providers at increased financial risk, may spur provider interest in patient engagement programs, including the use of decision and communication aids.

Finally, in some states, such as Washington State, health care reforms have included supporting “informed choice” or “informed consent” as state-level priorities.

### Limitations

There are several limitations to this study. The purchasers interviewed and surveyed are very large, with employees located throughout the U.S. and internationally. Being large, they tend to offer the richest benefits, and as such, represent a “high bar” for offerings and willingness to pay. Secondly, a relatively small sample of patients (14) were interviewed (likely those that were most motivated and interested in our study interventions), and their views including willingness to pay for decision and communication aids may not be representative of the larger population of patients with hip and knee OA. Finally, it is possible that despite the descriptions of decision and communication aids that were included in the surgeon survey, some surgeons may not be familiar with the terms, and therefore may have had difficulty rating their experience with and the value of these tools.

## Conclusions

In conclusion, we found widespread agreement among key stakeholders regarding the value of decision and communication aids, and relatively few barriers to adoption. The most significant barrier to adoption appears to be the cost of implementing these tools. Even there, we found some willingness to pay among patients and surgeons, while purchasers “passed the buck” on this issue to health plans. More work is needed to determine willingness to pay for decision and communication aids among patients, as our small sample suggested a bifurcation between patients who had no willingness to pay and who expressed a willingness to pay in the range of $50-$500 for the decision and communication aids they had recently experienced in our randomized controlled trial. Future studies should assess strategies for implementing these value-enhancing tools on a larger scale in the field of orthopaedic surgery.

## Electronic supplementary material

Additional file 1: **AAHKS MD Survey.** Copy of survey administered to surgeon members of AAHKS. (PDF 191 KB)
