# Supplementary material for: Patient, surgeon, and healthcare purchaser views on the use of decision and communication aids in orthopaedic surgery: a mixed methods study
Source: BMC Health Serv Res. 2014 Aug 31;14:366. doi: 10.1186/1472-6963-14-366 (PMC4162971; doi:10.1186/1472-6963-14-366)
Supplement: Supplementary file 1 — Additional file 1: AAHKS MD Survey. Copy of survey administered to surgeon members of AAHKS. (PDF 191 KB) [file 12913_2014_3468_MOESM1_ESM.pdf]

AAHKS
American Association of Hip and Knee Surgeons

2013 AAHKS Member Survey: Patient-Physician Shared Decision Making in Total Hip & Knee Arthroplasty

This is an ANONYMOUS 2-page survey designed to explore shared decision making preferences and practices in THA/TKA. Your complete responses are important. Please return BOTH PAGES of the questionnaire in the enclosed envelope or via fax to 847-698-0704 no later than April 30, 2013. Questions? Call the AAHKS at 847-698-1200.

1. Thinking about the patients you have seen in the last 12 months, to what extent does each of the following factors affect your recommendations regarding hip or knee replacement? Please rank the following patient factors from 1 thru 8, with "1" representing the factor that most strongly affects your recommendations, to "8" being the factor that least affects your recommendations:

- ☐ Physical exam findings (e.g., limp, pain with ROM, limited ROM)
- ☐ Patient's symptom severity
- ☐ Patient's values and preferences
- ☐ Degree of OA on radiographic images
- ☐ Patient's co-morbidities
- ☐ Patient's functional status
- ☐ We have exhausted conservative treatment options (Rx, Physical Therapy, etc.)
- ☐ Patient's age

2. "Routine practice" is defined as what you do more than half the time with your patients. Do you routinely (i.e., more than half the time) provide educational materials to potential THA/TKA patients before their initial office visit(s) that describe the benefits and risks of *surgical* options for treatment of advanced OA of the hip or knee? ○ Yes ○ No

3. Do you routinely (i.e., more than half the time) provide educational materials to potential THA/TKA patients before their initial office visit(s) that describe the benefits and risks of *non-surgical* options for treatment of advanced OA of the hip or knee? ○ Yes ○ No

4. If you routinely (i.e., more than half the time) provide educational materials to your hip/knee OA patients, how do you access these materials (please check all that apply)?

- ☐ Purchase directly from a vendor
- ☐ Provided by my medical group or health system
- ☐ Other (Please write your response in the box below)

5. Do you routinely (i.e., more than half the time) request that your patients develop a list of questions prior to their visit with you?
○ Yes ○ No

6. How do you assess your patients' preferences about surgical versus non-surgical treatment options (please describe)?

7. Do you routinely (i.e., more than half the time) provide your patients with any information that summarizes the discussion you had with them during their visit (e.g., an audio recording of their visit or dictated note)? ○ Yes ○ No

8. "Decision Aids" are defined as audio-visual or print materials that present benefits and risks of treatment options, including non-surgical options. Please select the appropriate response to describe your perception of the impact of Decision Aids on the following outcomes in your practice:

	Decrease	Neutral	Increase
a. Patient satisfaction	○	○	○
b. Your professional satisfaction	○	○	○
c. The quality of your interactions with patients	○	○	○
d. The frequency with which patients choose surgery	○	○	○
e. The volume of patients coming to your practice	○	○	○
f. Your total consultation time with the patient (possibly across more than 1 visit)	○	○	○
g. Your staff's total time interacting with the patient	○	○	○
h. Your practice costs	○	○	○

9. If your practice provided Decision Aids for all your potential hip/knee arthroplasty patients, how much would you estimate that this would increase your practice costs in dollars per patient (Please mark one selection):

○ $0-5 ○ $6-10 ○ $11-15 ○ Don't know ○ Other (please specify)

10. If this increased your practice costs per patient, how would your practice pay for this increment? (Please describe):

11. Would this intervention be something you would use in marketing to patients and payors about the quality of care you provide?
○ Yes ○ No

**12. If you have used Decision Aids with patients, did you:**	**Yes**	**No**	**N/A, did not use Decision Aids with patients**
a. Incur additional costs to your practice?	○	○	○
b. Bill for the process as a longer or extra appointment?	○	○	○
c. Get paid in some way for the time spent?	○	○	○

13. "Communication Aids" are defined as communication tools such as question lists, after visit summaries, and consultation audio recordings. Please select the appropriate response to describe your perception of the impact of Communication Aids on the following outcomes in your practice:

	Decrease	Neutral	Increase
a. Patient satisfaction	○	○	○
b. Your professional satisfaction	○	○	○
c. The quality of your interactions with patients	○	○	○
d. The frequency with which patients choose surgery	○	○	○
e. The volume of patients coming to your practice	○	○	○
f. Your total consultation time with the patient (possibly across more than 1 visit)	○	○	○
g. Your staff's total time interacting with the patient	○	○	○
h. Your practice costs	○	○	○

14. If your practice provided Communication Aids for all of your potential hip/knee arthroplasty patients, how much would your practice costs increase in dollars per patient? (Please mark one selection):

○ $0-5 ○ $6-10 ○ $11-15 ○ Don't know ○ Other (please specify) ☐

15. If this increased your practice costs per patient, how would your practice pay for this increment? (Please describe):

☐

16. Would this intervention be something you would use in marketing to patients and payors about the quality of care you provide?

○ Yes ○ No

17. If you have used Communication Aids with patients, did you:

	Yes	No	N/A, did not use Communication Aids with patients
a. Incur additional costs to your practice?	○	○	○
b. Bill for the process as a longer or extra appointment?	○	○	○
c. Get paid in some way for the time spent?	○	○	○

18. Imagine that a specific package of decision and communication aids was made available to your practice. Studies showed they had no effect on visit length, but led to more productive visits (e.g., patients reached an informed decision sooner, greater patient confidence in knowing what questions to ask, and surgeons felt that patients asked better questions). How many dollars per patient would you be willing to pay, out of your clinic budget, for your patients to access this package? (Please round to the nearest whole dollar) $ ☐

19. How would you expect to cover the added costs? (Please select all that apply):

☐ Increased productivity
☐ Increased reimbursement
☐ Increased volume of patients
☐ Other (please describe) ☐

20. Do you have an employee who helps you with education or counseling of patients? If yes, please state their title or job classification.

○ Yes ○ No ☐

21. My current practice setting is best described as (check one):

○ Academic Practice
○ Non-academic Hospital/HMO/Health System Employee
○ Military Practice (salary from military)
○ Private Practice - Orthopaedic Group
○ Private Practice - Multi-specialty Group
○ Other (please specify).. ☐

22. My practice is currently involved in (please check all that apply):

☐ Accepting bundled payments for surgeries
☐ Accepting "travel medicine" patients
☐ Part of an Accountable Care Organization (ACO)
☐ None of these

23. The revenues in my practice come in approximately the following percentages (please round to the nearest whole percent):

☐ % fee for service ☐ % capitated ☐ % bundled payment ☐ % other special reimbursement method

24. How many orthopaedists are in your practice? Please write in whole number. ☐

25. How many years have you been in practice? Please write in whole number. ☐

26. The state of your practice is located in: *2-letter abbreviation:* ☐☐

27. Did you complete an adult lower extremity reconstructive surgery fellowship? ○ Yes ○ No

28. Any other fellowship completed? ○ Yes ○ No **If yes, please describe:** ☐

29. What was your total THA/TKA surgery volume for the year 2012? Please write in whole number: ☐

30. Please estimate the percentage of types of cases you performed in 2012: Please write in whole %'s:

Primary THA: ☐ % Revision THA: ☐ % Primary TKA: ☐ % Revision TKA: ☐ %

Thank you for taking the time to complete this survey. Please return BOTH PAGES of the questionnaire in the enclosed envelope or via fax to 847-698-0704 no later than April 30, 2013. Questions? Call the AAHKS at 847-698-1200.
